# Empirical Study on the Transparency of Security Risk Information in Chinese Listed Pharmaceutical Enterprises Based on the ANP-DS Method

**DOI:** 10.1155/2020/4109354

**Published:** 2020-02-14

**Authors:** Jining Wang, Chong Guo, Tingqiang Chen

**Affiliations:** ^1^School of Economic and Management, Nanjing Tech University, Nanjing 211816, China; ^2^Research Center of Big Data Decision-Making and Social Performance Evaluation, Nanjing Tech University, Nanjing 211816, China

## Abstract

Frequent outbreaks of drug safety incidents pose a massive threat to public health and safety, while the transparency of security risk information in medical enterprises is not optimistic. Therefore, this study uses the analytic network process (Dempster-Shafer method) to construct a transparent comprehensive evaluation model for security risk information in listed pharmaceutical enterprises from the perspective of government supervision and listed pharmaceutical enterprises. On the basis of 59,305 data obtained by 303 enterprises listed in the Chinese biomedical sector, this research conducted an empirical study on the transparency of safety risk information in Chinese listed pharmaceutical enterprises. The current study found that the transparency of security risk information in Chinese listed pharmaceutical enterprises is generally between “general” and “relatively good” and tends to be “relatively good.” However, administrative punishment information, adverse drug reaction reporting systems, and production processes need continuous improvement.

## 1. Introduction

From the “Plum Blossom K” and “Guan Mu Tong” incidents to the Changchun Changsheng vaccine incident, these occurrences cause widespread concern for the pharmaceutical industry [1, 2] and seriously threaten the public's health and safety [3, 4], thereby affecting government credibility and social harmony and stability [5, 6]. Chen [7], Uthayakumar and Priyan [8], and de Korne et al. [9] believed that, as a field closely related to people's lives, health, and safety, medicines have a security risk in any part of the production, transportation, storage, and usage. Applequist et al. [10] and Listed [11] focused on the research and development of pharmaceuticals and study the entire pharmaceutical supply chain from the perspective of the full life cycle of drugs. They found risk, unique uncertainty, and other issues in the drug supply chain. Therefore, improving and strengthening the transparency of security risk information in listed pharmaceutical companies is imminent.

Drug safety incidents continue to occur. Davis [12], Naser [13], Zhang [14], Hossain [15], and Darmadi and Sodikin [16] conducted a series of studies to expose the causes of outbreaks. Ghorbel and Triki [17] find that companies have a good relationship with stakeholders to prove that their behavior is legal. However, the industry is characterized by substantial investment, high risk, and long industrialization cycle [18]. Operators use information asymmetry between consumers and business to maximize profits, fabricate production and product inspection records, and change process parameters and equipment arbitrarily [19, 20]. Chen et al. [21] pointed out that entrepreneurs reduce the sales cost to meet the consumer demand and to profit by maximizing their own interests and consumer utility because of moral considerations and other factors. Simultaneously, the imperfections of the drug regulatory system, backwardness of organizational concepts, and insufficiency of awareness on drug safety governance have resulted in the absence or lack of drug safety regulations [22]. Therefore, some scholars have explained that reducing the risk of drug safety entails drug regulatory authorities to enhance the forms and channels of information dissemination and improve the information disclosure, supervision, and evaluation system [23].

The world public medicine field is concerned with the reality of information transparency. The existing research focuses on the transparent supervision of drug information, transparency of the pharmaceutical industry chain in all aspects, and subject of participation [24–26]. For example, Vian et al. [27] found that the Medicines Transparency Alliance regulates the procurement of pharmaceuticals, drug policies, and drug supply, and establishes appropriate accountability in member states to promote transparency of drug information. Kaynak et al. [28] proposed that strengthening the transparency of the drug regulatory process can resolve such unethical behaviors as corruption in all aspects of the pharmaceutical industry chain. Formoso et al. [29] evaluated the primary sources of drug information in eight European countries and found that health professionals, policymakers, and the public are the main participants in drug information. Bushman and Smith [30] and Norris et al. [31] used corporate information transparency as research object and analyzed the factors affecting corporate information transparency from the perspective of government and industry. Chen et al. [32] introduced the SIRS contagion model of food safety risk considering the entry rate, the normal bankruptcy rate, the abnormal bankruptcy rate, and other correlated parameters of food enterprise, and they discussed and theoretically analyzed the influences of these correlated factors on the contagion of food safety risk. However, only a few studies have been conducted on the transparency of safety risk information in listed pharmaceutical companies, particularly the quantitative information disclosure with the characteristics of pharmaceutical companies. These studies have indicated the necessity to construct a transparency system of security risk information for listed pharmaceutical companies, quantify the safety risk information transparency of pharmaceutical companies, provide a useful reference model for security risk transparency in the pharmaceutical industry, and protect public health and safety.

The present study uses the preceding analysis as basis in using the Delphi method and network analytic network process (ANP) to construct an index system for judging the transparency of security risk information in medical enterprise from the perspective of government supervision and listed pharmaceutical enterprises. Moreover, this study uses the Dempster-Shafer evidence theory (DS) as basis in using Super Decisions and MATLAB to determine the weight of each index. The current study uses 59,305 data obtained from 303 enterprises listed in the Chinese biomedical sector to conduct an empirical study on the transparency level of security risk information for Chinese listed pharmaceutical companies.

The remainder of this paper is organized as follows.  Section 2 describes an evaluation index system for the transparency of security risk information in listed pharmaceutical companies.  Section 3 builds an evaluation model on the basis of ANP-DS.  Section 4 uses 59,305 data obtained from 303 enterprises listed in the Chinese biomedical sector to conduct an empirical study on the transparency level of security risk information for Chinese listed pharmaceutical companies.  Section 5 discusses and analyzes the results of empirical research. Lastly, Section 6 elaborates the conclusions.

## 2. Construction of an Evaluation Index System

The stakeholders of listed pharmaceutical enterprises obtain such information as financial status, business results, and social responsibility from outside the company. An operator's motivation, finances, environment, and traits determine the transparency of their security risk information [33–35]. Furthermore, the state forces listed enterprises to disclose their information to the public to improve the transparency of corporate security risk. However, the information disclosed by listed enterprises includes mandatory disclosure and voluntary disclosure [36]. Therefore, the present study constructs a transparent index system of security risk information in listed pharmaceutical companies from the perspective of government supervision and listed pharmaceutical enterprises to investigate the security risk information transparency of listed pharmaceutical companies.

### 2.1. Government Supervision Information Transparency

The government is the examination and approval party for drugs entering the market, makers of relevant laws and regulations, and supervisors of drug safety [1, 37]. Arnesano et al. [38] explained that government supervision of information transparency is the extent to which the government discloses their administration in the administrative process and the management of public information within the department or to the public at specified times and frequencies. Accordingly, improving the transparency of government supervision information for drugs can effectively realize the transparency of government regulatory information between governments and the government and the public, as well as eliminate public concerns on drug safety. The present study uses the literature and related laws as bases in analyzing the government supervision of information transparency from four aspects: safety supervision information, safety sampling information, clinical trial information, and accident emergency information.

#### 2.1.1. Safety Supervision Information

The government shoulders the responsibility of leading, directing, supervising, coordinating, safeguarding and guiding pharmaceutical matters within its administrative area to monitor drug safety in all aspects [1]. According to the existing achievements and Articles 72, 86, and 87 of the Drug Administration Law of the People's Republic of China, safety supervision information is divided into the following types: supervision focus, administrative punishment, risk warning, supervision announcement, production and business license directory, and media coverage [1, 39].

#### 2.1.2. Safety Sampling Information

Drug regulatory authorities regularly organize postmarket surveillance and sampling of biological products, including vaccines. For example, samples from market circulation are used to test the quality of vaccines [40] to reduce the safety risk of drugs. According to the existing achievements and Articles 24, 64, and 67 of the Drug Administration Law of the People's Republic of China, safety sampling information is divided into four third-level indexes: random inspection information, test instrument information, tracking inspection information, and chemical dangerous goods management information [41].

#### 2.1.3. Clinical Trial Information

Before the drugs are released to the market, relevant data and samples, such as research and development methods, quality indexes, and pharmacological and toxicological test results, must be submitted to the drug regulatory authority under the State Council. According to the existing research and Article 29 of the Drug Administration Law of the People's Republic of China, clinical trial information is measured from four aspects, namely, development method information, quality index information, pharmacological toxicology experimental results information, and clinical and nonclinical research quality management regulatory information [42, 43].

#### 2.1.4. Accident Emergency Information

The drug accident emergency and information linkage early warning mechanism can effectively prevent, control in a timely manner, and correctly dispose of drug quality accidents to protect the public's physical health and life safety [44]. According to the relevant literature and Article 70 of the Drug Administration Law of the People's Republic of China, the accident emergency information is divided into four third-level indexes: expired product recycling system, product return and recall system, product accident emergency mechanism, and adverse drug reaction reporting system [44, 45].

### 2.2. Listed Pharmaceutical Enterprises Information Transparency

If listed pharmaceutical enterprises can be self-disciplined, then they can effectively avoid the emergence of problematic products and strengthen their development as well. Self-discipline can promote the healthy development of the pharmaceutical industry through a virtuous circle, thereby protecting public health and safety [46]. According to the relevant literature and laws and regulations, the transparency information of listed pharmaceutical enterprises is divided into five second-level indexes: enterprise basic information, product information, safety production and sales information, enterprise governance information, and financial information [30, 46].

#### 2.2.1. Enterprise Basic Information

At present, the pharmaceutical industry has numerous manufacturers. Drug markets exist for illegal, low-level redundant construction; disordered market order; and consumers' single access to corporate information channels [47], which seriously affect the transparency of basic corporate information. Therefore, according to the existing research and Articles 7, 14, 42, and 70 of the Drug Administration Law of the People's Republic of China, the basic information of the enterprise is divided into four third-level indexes: pharmaceutical production license information, pharmaceutical business license information, basic situation of the Board of Supervisors members, and the integrity of other employees [48, 49].

#### 2.2.2. Product Information

Given the emergence of new sales channels, such as the Internet, global production and distribution channels have become complicated. Drug counterfeiters can enter the supply chain to sell products directly, thereby resulting in the need for product information transparency [50]. According to the existing achievements and the 31st, 54th, 60th, 61st, 62nd, and 6th chapters of the Drug Administration Law of the People's Republic of China, product information is divided into four third-level indexes: drug label and approval number, drug advertisement information, packaging materials and container information, and drug purchase inspection, acceptance, and custody information [50, 51].

#### 2.2.3. Safety Production and Sales Information

From research and development to final commercialization of biopharmaceuticals, basic research, pilot production, clinical trials, large-scale production, and marketization must be conducted [42]. If every link is guaranteed, then the quality of a drug can comply with the standard, protect public health and safety, and improve security risk transparency. According to the existing achievements and Articles 10, 15, 20, 26, and 56 of the Drug Administration Law of the People's Republic of China, safety production and sales information is divided into four third-level indexes: production process and records, equipment information, storage facilities, and purchase and sales records [42, 52].

#### 2.2.4. Enterprise Governance Information

The improvement of enterprise governance information transparency can improve government efficiency, correct violations of listed enterprises, enhance executive behavior constraints, and reduce agency costs [30, 53]. According to the current research and Article 18 of the Drug Administration Law of the People's Republic of China, the corporate governance framework, Board of Supervisors' decision-making supervision and performance, and enterprise governance information are analyzed from six aspects: enterprise governance framework, decision-making supervision and performance of the Board of Supervisors, reporting evaluations such as finance and social governance, rewards and punishments of the senior executive, participation of other stakeholders in governance, and equity concentration [53].

#### 2.2.5. Financial Information

The disclosure of information by listed enterprises mainly refers to the disclosure of finance, operation, and other aspects to the public under statutory or agreed requirements [54, 55]. The current study divides financial information into four third-level indexes: return on net assets, asset–liability ratio, operating cash flow, and growth rate of gross operating income [54, 55].

The correct selection and quantification of the evaluation indexes is the basis for the construction of an index system, which is also related to the merits of the evaluation results [56]. Moreover, following the basic principles of systemic, normative, and measurable in the process of constructing the evaluation index system is necessary. Therefore, this study comprehensively uses the Delphi expert survey method to demonstrate and improve the initially constructed index system. Lastly, an index system is formed to evaluate the transparency level of security risk information in Chinese listed pharmaceutical enterprises (Table 1 shows the specific indicator system). In particular, the Delphi expert group consists of 21 experts in pharmaceutical-related fields (i.e., 9 professors engaged in food and drug safety management research, 5 food and drug safety government regulatory authorities, 3 listed pharmaceutical company executives, 2 newspaper reporters involved in drug safety issues, and 2 ordinary consumers).

## 3. Evaluation Model

### 3.1. ANP-Based Index Weight Calculation

The analytic network process (ANP) is a scientific decision-making method based on the analytic hierarchy process (AHP) [57]. Nishizawa [58] believes that compared with AHP, ANP not only considers the hierarchical structure of the network but also considers the interaction and constraints between the indexes. Therefore, ANP can describe and characterize complex decision problems realistically.

In the network AHP, the entire decision system is divided into two parts, namely, the control and network layers. The decision criteria in the control layer are independent of each other and are typical AHP hierarchical structures [58]. Therefore, the weight of each decision criterion can be obtained by the traditional AHP method. Given that the different elements in the network layer interact with each other, an interactive network structure is formed [58]. The necessary steps are as follows.

  Step 1: construct a typical structure of ANP. First, build the control hierarchy and define the decision objectives and guidelines. Thereafter, calculate the weight of each decision criterion relative to the decisive goal. Lastly, analyze the interactions between the elements in each element set and build a network hierarchy.
  Step 2: construct a supermatrix to calculate the weights. Let the criteria corresponding to the target layer *G* in the control layer be *R*_1_, *R*_2_,…, *R*_*N*_, respectively, and the element set *E*_1_, *E*_2_,…, *E*_*N*_ exists in the network layer, whereas the element set *E*_*i*_ contains the elements *E*_*i*1_, *E*_*i*2_,…, *E*_*in*_, *i* = 1,2,…, *N*. Take the element *R*_*s*_ in the control layer as the criterion and the element *E*_*j*1_ in the element set *E*_*j*_ as the subcriterion. Analyze the importance of each element in the element set *E*_*j*_ and then construct the judgment matrix and obtain the normalized feature vector  (*w*_*i*1_, *w*_*i*2_,…,*w*_*in*_)^*T*^. That is, the network element sorting vector. Compared with the sorting vector of other elements, the supermatrix is obtained and marked as *w*_*ij*_.
  Step 3: construct a weighted supermatrix: Judge the importance of the elements *E*_1_, *E*_2_,… *E*_*N*_ for the criterion under the *R*_*s*_ criterion, and sorting by size, the normalized row vector is (*a*_1*j*_,…,*a*_*nj*_)^*T*^. The weighting matrix is $\;A=\left|\begin{array}{ccc} a_{11} & \cdots & a_{1N}\\ \cdots & \cdots & \cdots \\ a_{N1} & \cdots & a_{NN} \end{array}\right|$, constructing a weighted supermatrix of ANP on the basis of the preceding analysis $\bar{w}=a_{ij}w_{ij}$.
  Step 4: extreme supermatrix $\underset{k⟶\infty }{\lim}w^{k}$. On the basis of constructing a weighted supermatrix, we stable processing *W*. That is, calculating the limit relative sorting vector: $\underset{N⟶\infty }{\lim}\left(1/N\right)\sum_{k=1}^{N}W^{k}$. If the limit converges and is unique, then the relative ordering of the elements in the network layer under the criterion layer for the element *j* is the *j*th column of *W*^*∞*^. That is, the weight value of each element relative to the ultimate goal.

### 3.2. Evaluation Model for the Transparency of Security Risk Information in Listed Pharmaceutical Enterprises Based on DS

In the evaluation, numerous information show different degrees of ambiguity, and evidence theory can better describe the uncertainty in decision problems [59]. By contrast, the Dempster–Shafer synthesis formula combines different reliability functions that can integrate the opinions of decision-makers effectively [60]. Therefore, a comprehensive evaluation model based on evidence theory was finally established.

#### 3.2.1. Evaluation Index Weights and Evaluation Sets

In this section, the method for determining the weight of each index is ANP and the weights of the third-level indexes *P*, *P*_*i*_ and *P*_*ij*_ are represented by *λ*, *λ*_*i*_, and *λ*_*ij*_. The weight values of the layers are normalized using the method of *λ*_*i*_/max(*λ*_*i*_) or *λ*_*ij*_/max(*λ*_*ij*_) [60], and according to the expert's preference coefficient *a*(0.9 ≤ *a* ≤ 1), adjust the basic trust distribution function as follows:

(1) $$\begin{array}{c} M_{i}^{\prime }\left(A_{i}\right)=\frac{\lambda_{i}}{\max\left(\lambda_{i}\right)}\cdot a\cdot M_{i}^{\prime \prime }\left(A_{i}\right)\\ \text{or }M_{i}^{\prime }\left(A_{i}\right)=\frac{\lambda_{ij}}{\max\left(\lambda_{ij}\right)}\cdot a\cdot M_{i}^{\prime \prime }\left(A_{i}\right). \end{array}$$

Assume that its rating is *H*={*H*_1_, *H*_2_, *H*_3_, *H*_4_, *H*_5_}, which means the transparency evaluation of security risk information in Chinese listed pharmaceutical enterprises is good, relatively good, average, relatively poor, and poor. Simultaneously, the evaluation value *V*_*i*_ of each transparency level *H*_*i*_ is set in advance on the basis of the characteristics of information transparency.

#### 3.2.2. Building Basic Credibility Allocation

Evidence theory indicates that the transparency evaluation of security risk information in Chinese listed pharmaceutical enterprises under different levels of third-level indexes is considered to be the lowest subproposition [59], and confidence is directly assigned to the different performance levels by the evaluation experts. If there are *q* experts who evaluate the security risk information transparency, then *p*_*ij*_(*H*_*h*_, *p*) indicates the confidence level of the expert *p* on the information transparency level of the index *c*_*ij*_. The largest index of weight for each index set is the key index, whereas the others are nonkey indexes [60]. Assume that *c*_*l*_ is the third-level index with the highest weight under the first-level index *C*, and its basic credibility is assigned as follows:

(2) $$\begin{array}{c} m\left(\left.H_{h}\right|c_{l}\right)=\alpha_{l}^{p}P_{l}\left(H_{h},p\right),\\ m\left(\left.H_{\Theta }\right|c_{l}\right)=1-\overset{4}{\underset{h=1}{\sum }}m\left(\left.H_{h}\right|c_{l}\right), \end{array}$$

where *m*(*H*_Θ_|*c*_*l*_) represents a completely unascertained basic credibility assignment and *α*_*l*_^*p*^ is the preference coefficient of expert *p* for the key subindex *c*_*l*_ in *C* [61], and its value is [0.9, 1]. Moreover, the larger the value, the more important the key subindexes and the nonkey subindexes.

### 3.3. Synthesis of the ANP-DS Calculation Results

#### 3.3.1. Transparency of Security Risk Information in Listed Pharmaceutical Enterprises Based on the ANP-DS Model

The basic credibility of the third-level index is allocated to synthesize the basic credibility of the third-level index *c*_*ij*_ via the *q* expert(s), which can form a matrix $m\left(i,j\right)=\left[\begin{array}{cccc} m_{10} & m_{11} & \cdots & m_{1h}\\ m_{20} & m_{21} & \cdots & m_{2h}\\ \vdots & \vdots & \vdots & \vdots \\ m_{q0} & m_{q1} & \cdots & m_{qh} \end{array}\right]$, while *m*_*p*0_=*m*_*p*_(*H*_Θ_|*c*_*ij*_), *m*_*ph*_=*m*_*p*_(*H*_*h*_|*c*_*ij*_). A recursive algorithm is used to avoid the intersection calculation and to synthesize the evidence for the basic credibility of the third-level index *c*_*ij*_ by *q* expert(s) [61]. Assume that the first *r* expert(s) information sets are *I*(*r*)={1,2,…, *i*},  1 ≤ *r* ≤ *q*. The *r* basic credibility of the first *r* rows in the matrix *m*(*i*, *j*) is assigned as *m*_*I*(*r*),0_=*m*_*I*(*r*)_(*s*_Θ_|*c*_*ij*_), *m*_*I*(*r*),*h*_=*m*_*I*(*r*)_(*s*_*h*_|*c*_*ij*_).

#### 3.3.2. Calculate the Transparency Level of Trust in the Security Risk Information for Listed Pharmaceutical Enterprises on the Basis of ANP-DS

The reliability and likelihood of each rating of the transparency for the listed pharmaceutical enterprises in security risk information [59] are as follows:

(3) $$\begin{array}{c} \text{Bel}\left(H_{i}\right)=m\left(H_{i}\right),\text{Pl}\left(H_{i}\right),\text{Pl}\left(H_{i}\right)=1-\text{Bel}\left(\bar{H_{i}}\right). \end{array}$$

Equation (3) can obtain a confidence interval for each evaluation level [Bel(*H*_*i*_), Pl(*H*_*i*_)]. According to the confidence interval, the trust degree is *E*(*H*_*i*_)=Bel(*H*_*i*_)+[1 − (Pl(*H*_*i*_) − Bel(*H*_*i*_))](Pl(*H*_*i*_) − Bel(*H*_*i*_)).

In particular, Pl(*H*_*i*_) − Bel(*H*_*i*_) indicates that the evidence supports the uncertainty of information transparency as *H*_*i*_. The grades with the highest degree of trust in various evaluation levels correspond to the comprehensive evaluation of listed pharmaceutical enterprises security risk information transparency.

#### 3.3.3. Comprehensive Evaluation Value Calculation in the Transparency of Security Risk Information for Listed Pharmaceutical Enterprises Based on the ANP-DS Model

Assume that the evaluation value of the information transparency evaluation level *H*_*i*_ is *V*_*i*_, while the evaluation value of the transparency of the first-level index *S* information can be obtained as *Q*: *Q*=∑_*h*=1_^5^*V*_*h*_*∗m*(*H*_*h*_|*S*). The evaluation effect of information transparency can be obtained through the above analysis of listed pharmaceutical enterprises security risk information transparency.

## 4. Empirical Research on the Transparency of Security Risk Information in Listed Pharmaceutical Enterprises Based on ANP-DS

### 4.1. Establish an Evaluation Model

**Figure 1:**
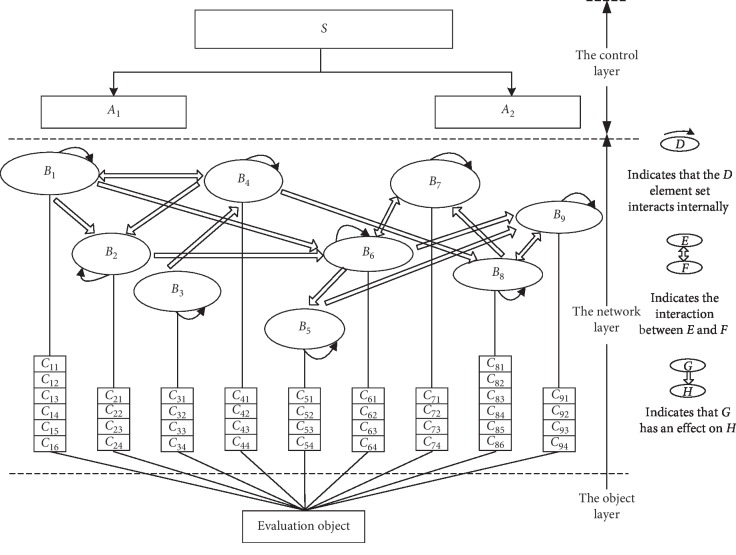
Model structure of transparency for listed pharmaceutical enterprises in security risk information.

In the constructed evaluation index system, the indexes within the standard layer are not independent of one another, although interactions are evident. For example, the safety supervision information and accident emergency information have an interaction. Therefore, the elements of the evaluation indexes and internal factors of the elements will inevitably interact rather than be independent. That is, the relationship between the indexes in the index system should be a network relationship, rather than a simple hierarchical relationship [62]. The preceding analysis and structure of the ANP model indicate that the present study constructs the network structure of the evaluation model (see Figure 1 for the model structure).

### 4.2. Determination of Index Weight

The process of determining the weight of an index using the ANP method is the same as that of the AHP. Judgment regarding the importance of each index according to the expert is necessary, and then a judgment matrix is constructed to determine the weight of each index. To ensure the rationality of the index weights, an expert group consisting of 11 experts was invited to judge the importance of each index. At the same time, we developed the pairwise comparison matrices based on the ANP model [63]. The relative importance values are determined on a scale of 1–9 [57, 63, 64], where 1 means equal importance between the two elements and 9 indicates the extreme importance of one element compared with the other one (see Table 2). In the current study, the data in the recycled Delphi expert questionnaire are used and combined with the principle of ANP. The local weight and global weight of each index are calculated via Super Decisions software.

#### 4.2.1. Weight of Internal Independent Index Layer

In the ANP model, the 1–9 scaling method of Saaty is used to represent the importance of the factors, and the judgment matrix is expressed by the interval number [63]. Because A_1_ and A_2_ are independent indicators, there is no need to consider the interaction between factors, and the eigenvector method can be used to determine index weights. We used the data from the three-round Delphi expert questionnaire, construct a judgment matrix between the two first-level indexes, and derive the weights of the first-level indexes (see Table 3).

#### 4.2.2. Consistency Test

When CR ≤ 0.1, the judgment matrix can be considered to have satisfactory consistency. The random consistency ratio is first tested to ensure the reliability of the construction of the index system and the rationality of the expert scoring. Through the consistency test scores of each judgment matrix in Table 4, the judgment matrix constructed by the expert scoring system has passed the consistency test and has satisfactory consistency under the index system constructed in this chapter. The judgment matrix also explains the scientificity and rationality of the weights obtained in this chapter.

#### 4.2.3. Reliability and Validity Test

This study conducted a reliability and validity test to test the credibility and validity of the survey results, as presented in Table 5:

As can be seen from Table 5, Cronbach's alpha value of government supervision and enterprise reliability index is above 0.9, indicating that the transparency index measurement of government regulation and enterprise information have good internal consistency and stability, and the reliability is good. The validity index of KMO is above 0.9, indicating that the factor analysis is suitable and the validity is high.

#### 4.2.4. Network Layer Index Set and Weight of Each Index

The set of indexes of the network layer and indexes are not entirely independent and have a certain influence on each other. Therefore, when calculating the index weight, the local weight and global weight are obtained via the ANP method strictly according to the network hierarchy.

Firstly, we get the local weight of Bi through the interval judgment matrix constructed by experts (see Table 6).

To alleviate the mathematical burden, the following calculations were implemented through the software Super Decisions [65]. On completion of all pairwise comparison matrices, the unweighted supermatrix is built based on the interrelationship of each index in the Bi network layer (Table 7).

Then, we used the “Computations/Weighted Super Matrix” command in Super Decisions software and obtained the weighted supermatrix of the Bi evaluation index of the transparency for listed pharmaceutical enterprises in security risk information (Table 8).

Lastly, raising the weighted supermatrix to limiting powers until the weights converge and remain stable, the limit supermatrix will be achieved. At the same time, we got the global weight of Bi. The global weight of Ci can also be obtained through the same steps. Based on the above calculation steps, we can get the specific weight of each index of the transparency for listed pharmaceutical enterprises in security risk information (Table 9 shows the specific weight of each index).

### 4.3. Sample and Data Characteristics

The current study selected 303 listed enterprises in the biomedical sector as samples by consulting the Ruisi database. According to the indexes built in the previous section, the corresponding scoring standards are designed and divided into five categories: “good,” “relatively good,” “general,” “relatively poor,” and “poor.” Lastly, the evaluation criteria for the transparency score of security risk information in listed pharmaceutical enterprises are as follows: 100 points for full marks; 60, passing; 0–30, poor; 30–60, relatively poor; 60–75, general; 75–90, relatively good; and 90–100, good. We provide professional knowledge training for each investigator to collect sample information objectively and accurately. We modify and improve the sample form through pre-acquisition to determine the final sampling form for the transparent investigation of safety risk information in listed pharmaceutical enterprises.

According to the data collection form developed, we collected data in January and February 2019 through the websites of the government, enterprises, and China Securities Regulatory Commission and obtained 59,305 relevant data for transparency of security risk information in listed pharmaceutical enterprises. In this research, the sample is divided into 7 categories according to the economic region where the enterprise is located. The data samples are mainly concentrated in North and Southern China, with a total of 172 samples, and account for 56.77% of the total sample. Compared with the developed coastal areas, the sample size data of the Northeast and Northwest China are relatively small, and the number of enterprises is 8.91% of the sample size, which is below 10%.  Table 10 shows the data characteristics.

### 4.4. Comprehensive Evaluation Value Calculation for Transparency of Security Risk Information in Listed Pharmaceutical Enterprises Based on the ANP-DS Model

According to the composition of the indexes for the transparency of security risk information in listed pharmaceutical enterprises (see Table 1), 11 experts from the drug safety experts, the market supervision administration department, and the marketing department of the listed biomedical enterprises were invited to analyze the relevant materials and data. Furthermore, experts have given confidence to the transparency evaluation indicators of listed pharmaceutical companies' safety risk information.

Suppose *H* = {good (*H*_1_), relatively good (*H*_2_), general (*H*_3_), relatively poor (*H*_4_), poor (*H*_5_)} is the fuzzy evaluation set selected for this section, and its fuzzy evaluation reference value is *p*(*H*)={*p*(*H*_1_),  *p*(*H*_2_),  *p*(*H*_3_),  *p*(*H*_4_), *p*(*H*_5_)}={0.95, 0.8, 0.6, 0.4, 0.2}. We assume that the decision maker's preference coefficient *α* = 0.9, and normalize the indexes of each layer in Table 9 and the normalized calculation results based on the local weight calculation are revealed in Table 11.

According to the survey and statistical results of the external and internal indexes, respectively, experts gave the initial evidence credibility regarding the third-level indexes for the transparency of security risk information in listed pharmaceutical enterprises after discussion. Initial credibility is multiplied by the weight corresponding to each third-level index. That is, the basic credibility calculation result of the third-level indicator is obtained (Appendix Table 3 presents the specific data). In the DS synthesis process, the current study uses the steps of evidence theory evaluation to write the DS synthesis program using MATLAB, owing to the extensive calculation. Using this program, we synthesize the basic credibility of the third-level indexes and obtain the initial credibility of the second-level indexes (the specific data is in Appendix Table 4). In the same manner, the basic credibility calculation results of the second-level indexes and initial credibility calculation results of the first-level indexes are obtained (specific data are presented in Appendix Table 5 and Appendix Table 6). Lastly, we use the programmed procedure to synthesize the results of the basic credibility of the first-level indexes (Appendix Table 7 provides the specific data) and obtain the credibility of the transparency level for the security risk information in listed pharmaceutical enterprises (see Table 12).

According to the formula, the comprehensive evaluation results of transparency for security risk information in Chinese listed pharmaceutical enterprises are as follows:

(4) $$\begin{array}{c} H=\overset{5}{\underset{h=1}{\sum }}V_{h}*m\left(\left.H_{h}\right|C\right)=0.0241\times 0.95+0.6728\times 0.8+0.2890\times 0.6+0.0032\times 0.4+0.0004\times 0.2=0.7360. \end{array}$$

For the transparency of security risk information in Chinese listed pharmaceutical enterprises, due to 0.60 < 0.7360 < 0.80, the transparency level is between “general” and “relatively good,” and is inclined to be “relatively good.”

## 5. Empirical Results

Through the transparency evaluation of security risk information in Chinese listed pharmaceutical enterprises based on the ANP-DS model, we find that the reasons for the transparency of security risk information in Chinese listed pharmaceutical enterprises are as follows.

1. Among the 40 indexes for evaluating the transparency of safety risk information in Chinese listed pharmaceutical enterprises, a total of 3 indexes are at the levels of “relatively good” and “good” (Table 13).

  Compared with other indexes, the information transparency of the indexes in Table 13 is above 0.8, which is between “relatively good” and “good.”
(2) Among the 40 indexes for evaluating the transparency of safety risk information in Chinese listed pharmaceutical enterprises, 34 indexes are at the level between “general” and “relatively good” (Table 14).

  The evaluation level of the indexes in Table 14 is between “general” and “relatively good.” The reason for the problems of these indexes is first the lack of legal links. The existing “Drug Administration Law of the People's Republic of China,” “Regulations on the Implementation of the Drug Administration Law of the People's Republic of China,” and “Regulations on the Administration of Drug Quality Supervision and Sampling,” as well as other provisions of the law, have further improved the Chinese drug safety legal system. However, during the review process from the production to the circulation of the drug, if the auditor violates the law and regulations, then the matter of the disposal of the certificate issued by the auditor and the relevant person responsible for the enterprise has no corresponding regulation.
  The ethics of pharmaceutical enterprises are lacking. The approval of the drug is based on the fact that the application materials and samples submitted by the enterprise are accurate and reliable, but the quality of the product cannot be guaranteed. Furthermore, enterprises directly tamper with production quality data and forge production and inspection records to reduce costs and obtain high profits.
  Government supervision is considerably lacking. The institutions responsible for reviewing and supervising drugs are complicated, including the Drug Evaluation Center of the General Administration, Food and Drug Inspection and Inspection Center of the General Administration, and Chinese Food and Drug Testing Institution. This decentralization of power has led to an inconsistent division of powers and responsibilities, resulting in a long-term situation of “separate government” in the drug regulatory authorities and contributed to the expansion of the drug incident. Moreover, current drug supervision is a mere formality. For example, local area owns an abnormal reaction investigation agency for disease prevention and control, but the agency undertakes the amount of vaccination work.
  In addition, third-party participation in drug safety is low. The supervision of pharmaceutical enterprises is affected by such factors as policy burden and regulatory capture and relies on the government. Moreover, the consumer, media, and pharmaceutical industry associations have a weak sense of responsibility, and the situation of coordinated management by government, enterprises, and third parties has yet to be formed.
(3) Among the 40 indexes for evaluating the transparency of safety risk information in Chinese listed pharmaceutical enterprises, 3 indexes are at the level between “relatively poor” and “general” (Table 15).

The current design of the adverse drug reaction reporting system is flawed. The reporting methods are spontaneous and nonmandatory and rely heavily on the subjective judgment and reporting effort of the reporter. These factors lead to the low availability of adverse drug reaction cases in China, which seriously affects the early warning ability of drug safety. Furthermore, the processes of discovery, reporting, verification, evaluation, and risk control in the adverse drug reaction monitoring system involve multiple parties. For example, the drug regulatory and health departments are the competent authorities for monitoring and reporting in the adverse drug reaction monitoring system. However, the two government departments are not affiliated with each other, and simultaneously have cross-cutting in drug regulatory aspects of medical institutions. The drug regulatory authorities need to seek the opinions of the health administrative department when formulating relevant policies. Management departments are different; however, they have conflicts of interest, and therefore can shirk their responsibilities.

The new version of Good Manufacturing Practice (GMP) has raised the requirements for pharmaceutical enterprises in terms of equipment, production environment and conditions, production processes, management systems, and other aspects. These requirements can assist in controlling the quality and safety of the drug from the source and ensuring the safety, effectiveness, and uniformity of the drug quality. However, after the completion of the certification, some pharmaceutical manufacturers have deliberately relaxed the quality control from the factory entrance of the raw materials to the final product for cost reduction, sacrificed the quality of the drugs, or violated and illegally produced the requirements of the new GMP. Moreover, the production process is often not kept a secret, and the pharmaceutical enterprise falsely states the process or deliberately submits a registration process with incomplete and fuzzy parameters. In addition, enterprises intentionally exclude the deviation of the quality management of the enterprise's products to reduce the impact on the GMP review results, causing the actual production process of the drug to be hidden from the preapproval process and not be traced when drug safety problems occur.

The “Regulations on Administrative Punishment Procedures for Drug Administration” in the field of drug regulation in China has not yet accurately defined various concepts. In the process of administrative law enforcement, an inevitable fuzzy zone exists in the application of the law, and the understanding between the administrative organs and the administrative counterparts is not synchronized. The imperfection of the drug administrative law enforcement suspected criminal case transfer system has led to a certain lag in consumers' administrative punishment information. Therefore, the transparency of this index is low.

## 6. Conclusion

In recent years, the pressure of public opinion in the Chinese pharmaceutical industry has remained high, and corporate brands and reputation have been seriously damaged. Scholars actively explore how to block loopholes, eliminate risk, protect public health and safety, and improve the transparency of safety risk information in pharmaceutical companies. This study uses the ANP-DS method to construct a transparent comprehensive evaluation model for security risk information in listed pharmaceutical enterprises from the perspective of government supervision and listed pharmaceutical enterprises. Based on the 59,305 data obtained via 303 listed enterprises in the Chinese biomedical sector, we conducted an empirical study on the transparency of security risk information in Chinese listed pharmaceutical enterprises. The research conclusions are as follows.

1. The overall transparency level of the security risk information in Chinese listed pharmaceutical enterprises is between “general” and “relatively good” and tends to be “relatively good.” The overall transparency level has not yet reached the “good” level, and a certain gap continues to exist.
2. The transparency of government supervision information is between “general” and “relatively good,” but biased toward the “general.” The main reason is that in view of the problems in drug practice, blank and fuzzy areas continue to exist in the laws and regulations related to drug supervision. In particular, the drug reaction reporting system and administrative punishment information have seriously affected the transparency of government supervision information.
3. The transparency of listed pharmaceutical enterprises information is between “general” and “relatively good” and tends to be “relatively good.” However, a room for improvement in the production process and recorded information of enterprises continues.

The research results have specific reference significance for managing listed pharmaceutical enterprises and protecting consumer rights. However, this research only selects the data of the past three years and does not adequately consider the persistent impact of specific indicators on the transparency of the security risk information of listed pharmaceutical companies. Succeeding research can supply certain dynamic indexes to improve the existing index system and conduct a large sample of data on safety risk information of listed pharmaceutical enterprises in China for nearly 10 years, thereby making the results increasingly accurate and reliable.

## Figures and Tables

**Table 1:** Index system for the transparency of security risk information in Chinese listed pharmaceutical enterprises.

Objective	First-level index	Second-level index	Third-level index
Transparency of security risk information in Chinese listed pharmaceutical enterprises (*S*)	Government supervision information transparency (*A*_1_)	Safety supervision information (*B*_1_)	Supervision focus information (*C*_11_)
			Administrative punishment information (*C*_12_)
			Risk warning information (*C*_13_)
			Supervision announcement information (*C*_14_)
			Production and business license directory information (*C*_15_)
			Media coverage information (*C*_16_)
		Safety sampling information (*B*_2_)	Random inspection information (*C*_21_)
			Test instrument information (*C*_22_)
			Tracking inspection information (*C*_23_)
			Chemical dangerous goods management information (*C*_24_)
		Clinical trial information (*B*_3_)	Development method information (*C*_31_)
			Quality index information (*C*_32_)
			Pharmacological toxicology experimental results information (*C*_33_)
			Clinical and nonclinical research quality management regulatory information (*C*_34_)
		Accident emergency information (*B*_4_)	Expired product recycling system (*C*_41_)
			Product return and recall system (*C*_42_)
			Product accident emergency mechanism (*C*_43_)
			Adverse drug reaction reporting system (*C*_44_)
	Listed pharmaceutical enterprises information transparency (*A*_2_)	Enterprise basic information (*B*_5_)	Pharmaceutical production license information (*C*_51_)
			Pharmaceutical business license information (*C*_52_)
			Basic situation of the board of supervisors members (*C*_53_)
			The integrity of other employees (*C*_54_)
		Product information (*B*_6_)	Drug label and approval number (*C*_61_)
			Drug advertisement information (*C*_62_)
			Packaging materials and container information (*C*_63_)
			Drug purchase inspection, acceptance and custody information (*C*_64_)
		Safety production and sales information (*B*_7_)	Production process and records (*C*_71_)
			Equipment information (*C*_72_)
			Storage facilities (*C*_73_)
			Purchase and sales records (*C*_74_)
		Enterprise governance information (*B*_8_)	Enterprise governance framework (*C*_81_)
			Decision-making supervision and performance of the Board of Supervisors (*C*_82_)
			Reporting evaluations such as finance and social governance (*C*_83_)
			Rewards and punishments of the senior executive (*C*_84_)
			Participation of other stakeholders in governance (*C*_85_)
			Equity concentration (*C*_86_)
		Financial information (*B*_9_)	Return on net assets (*C*_91_)
			Asset-liability ratio (*C*_92_)
			Operating cash flow (*C*_93_)
			Growth rate of gross operating income (*C*_94_)

**Table 2:** Relative importance scale.

Scale	Definition
1	Equally important
3	Moderately more important
5	Strongly more important
7	Very strongly more important
9	Extremely more important
2, 4, 6, 8	Mean intermediate values

**Table 3:** Judgment matrix of the control layer of transparency for listed pharmaceutical enterprises in security risk Information.

*S*	*A*_1_	*A*_2_	Weight
*A*_1_	1	2	0.667
*A*_2_	1/2	1	0.333
Comment: CR = 0.000 < 0.1			

**Table 4:** Summary of the consistency test scores of the judgment matrix.

Judgment matrix	CR value	Judgment matrix	CR value	Judgment matrix	CR value
*S* ⟶ *A*	0.000	*A*_1_ ⟶ *B*	0.000	*B*_1_ ⟶ *C*	0.000
				*B*_2_ ⟶ *C*	0.000
				*B*_3_ ⟶ *C*	0.000
				*B*_4_ ⟶ *C*	0.006
		A_2_ ⟶ *B*	0.000	*B*_5_ ⟶ *C*	0.000
				*B*_6_ ⟶ *C*	0.009
				*B*_7_ ⟶ *C*	0.000
				*B*_8_ ⟶ *C*	0.006
				*B*_9_ ⟶ *C*	0.000

**Table 5:** Reliability and validity test of the survey sampling table.

Survey sample subject	Cronbach's alpha	KMO	Bartlett	df	Sig
Government	0.921	0.926	15260.314	65	0.000
Enterprise	0.904	0.917	5613.276	24	0.000

**Table 6:** Judgment matrix of the Bi of transparency for listed pharmaceutical enterprises in security risk Information.

*B*	*B*_1_	*B*_2_	*B*_3_	*B*_4_	*B*_5_	*B*_6_	*B*_7_	*B*_8_	*B*_9_	Local weight
*B*_1_	1	3	2	1						0.351
*B*_2_	1/3	1	2	3						0.110
*B*_3_	1/2	1/2	1	2						0.189
*B*_4_	1	1/3	1/2	1						0.351
*B*_5_					1	1	2	2	2	0.277
*B*_6_					1	1	2	3	3	0.328
*B*_7_					1/2	1/2	1	1	1	0.139
*B*_8_					1/2	1/3	1	1	1	0.128
*B*_9_					1/2	1/3	1	1	1	0.128

**Table 7:** Unweighted supermatrix for the Bi network layer.

Unweighted supermatrix		*B*								
		*B*_1_	*B*_2_	*B*_3_	*B*_4_	*B*_5_	*B*_6_	*B*_7_	*B*_8_	*B*_9_
*B*	*B*_1_	0.351	0.338	0.000	0.444	0.000	0.000	0.000	0.000	0.000
	*B*_2_	0.089	0.090	0.000	0.000	0.000	0.000	0.000	0.000	0.000
	*B*_3_	0.000	0.000	0.286	0.000	0.000	0.000	0.000	0.000	0.000
	*B*_4_	0.317	0.320	0.571	0.444	0.000	0.000	0.000	0.000	0.000
	*B*_5_	0.000	0.000	0.000	1.000	0.667	0.000	0.000	0.000	0.000
	*B*_6_	0.183	0.169	0.000	0.000	0.000	0.467	0.000	0.000	0.000
	*B*_7_	0.000	0.094	0.143	0.000	0.000	0.191	0.500	0.000	0.000
	*B*_8_	0.078	0.000	0.000	0.111	0.000	0.171	0.500	0.500	0.000
	*B*_9_	0.000	0.000	0.000	0.000	0.333	0.171	0.000	0.500	1.000

**Table 8:** Weighted supermatrix for the Bi network layer.

Weighted supermatrix		*B*								
		*B*_1_	*B*_2_	*B*_3_	*B*_4_	*B*_5_	*B*_6_	*B*_7_	*B*_8_	*B*_9_
*B*	*B*_1_	0.167	0.169	0.000	0.222	0.000	0.000	0.000	0.000	0.000
	*B*_2_	0.044	0.045	0.000	0.000	0.000	0.000	0.000	0.000	0.000
	*B*_3_	0.000	0.000	0.143	0.000	0.000	0.000	0.000	0.000	0.000
	*B*_4_	0.158	0.160	0.286	0.222	0.000	0.000	0.000	0.000	0.000
	*B*_5_	0.000	0.000	0.000	0.000	0.333	0.000	0.000	0.000	0.000
	*B*_6_	0.091	0.169	0.000	0.000	0.000	0.233	0.000	0.000	0.000
	*B*_7_	0.000	0.084	0.071	0.000	0.000	0.095	0.250	0.000	0.000
	*B*_8_	0.039	0.042	0.000	0.056	0.000	0.086	0.250	0.250	0.000
	*B*_9_	0.000	0.000	0.000	0.000	0.167	0.086	0.000	0.250	0.500

**Table 9:** Weight of the evaluation index system.

First-level index	Weight	Second-level index	Local weight	Global weight	Third-level index	Local weight	Global weight
*A*_1_	0.667	*B*_1_	0.351	0.216	C_11_	0.167	0.030
					*C*_12_	0.167	0.030
					*C*_13_	0.341	0.061
					*C*_14_	0.141	0.025
					*C*_15_	0.084	0.015
					*C*_16_	0.100	0.018
		*B*_2_	0.109	0.045	*C*_21_	0.280	0.010
					*C*_22_	0.127	0.005
					*C*_23_	0.312	0.012
					*C*_24_	0.280	0.010
		*B*_3_	0.189	0.027	*C*_31_	0.250	0.005
					*C*_32_	0.250	0.005
					*C*_33_	0.250	0.005
					*C*_34_	0.250	0.005
		*B*_4_	0.351	0.189	*C*_41_	0.250	0.039
					*C*_42_	0.250	0.039
					*C*_43_	0.250	0.039
					*C*_44_	0.250	0.039

*A*_2_	0.333	*B*_5_	0.277	0.020	*C*_51_	0.250	0.004
					*C*_52_	0.250	0.004
					*C*_53_	0.250	0.004
					*C*_54_	0.250	0.004
		*B*_6_	0.328	0.092	*C*_61_	0.500	0.038
					*C*_62_	0.167	0.013
					*C*_63_	0.167	0.013
					*C*_64_	0.167	0.013
		*B*_7_	0.139	0.050	*C*_71_	0.250	0.010
					*C*_72_	0.250	0.010
					*C*_73_	0.250	0.010
					*C*_74_	0.250	0.010
		*B*_8_	0.128	0.152	*C*_81_	0.110	0.014
					*C*_82_	0.220	0.028
					*C*_83_	0.220	0.028
					*C*_84_	0.237	0.030
					*C*_85_	0.103	0.013
					*C*_86_	0.110	0.014
		*B*_9_	0.128	0.211	*C*_91_	0.250	0.087
					*C*_92_	0.250	0.087
					*C*_93_	0.250	0.087
					*C*_94_	0.250	0.087

**Table 10:** Distribution of data collection samples of listed pharmaceutical enterprises.

	Eastern China	Northeast China	North China	Central China	Southern China	Southwest China	Northwest China
Quantity	37	17	111	35	61	32	10
Proportion	0.122	0.056	0.366	0.116	0.201	0.106	0.033

**Table 11:** Standardized value of the index evaluation weight.

First-level index	Weight	Second-level index	Weight	Third-level index	Weight
*A*_1_	0.950	*B*_1_	0.950	*C*_11_	0.465
				*C*_12_	0.465
				*C*_13_	0.950
				*C*_14_	0.393
				*C*_15_	0.233
				*C*_16_	0.278
		*B*_2_	0.196	*C*_21_	0.854
				*C*_22_	0.388
				*C*_23_	0.950
				*C*_24_	0.854
		*B*_3_	0.117	*C*_31_	0.950
				*C*_32_	0.950
				*C*_33_	0.950
				*C*_34_	0.950
		*B*_4_	0.828	*C*_41_	0.950
				*C*_42_	0.950
				*C*_43_	0.950
				*C*_44_	0.950

*A*_2_	0.475	*B*_5_	0.088	*C*_51_	0.950
				*C*_52_	0.950
				*C*_53_	0.950
				*C*_54_	0.950
		*B*_6_	0.414	*C*_61_	0.950
				*C*_62_	0.317
				*C*_63_	0.317
				*C*_64_	0.317
		*B*_7_	0.224	*C*_71_	0.950
				*C*_72_	0.950
				*C*_73_	0.950
				*C*_74_	0.950
		*B*_8_	0.684	*C*_81_	0.441
				*C*_82_	0.881
				*C*_83_	0.881
				*C*_84_	0.950
				*C*_85_	0.414
				*C*_86_	0.441
		*B*_9_	0.950	*C*_91_	0.950
				*C*_92_	0.950
				*C*_93_	0.950
				*C*_94_	0.950

**Table 12:** Credibility of transparency for security risk information in listed pharmaceutical enterprises index evaluation.

	Good	Relatively good	General	Relatively poor	Poor
Confidence	0.0241	0.6728	0.289	0.0032	0.0006

**Table 13:** Indexes and scores for the level between “relatively good” and “good” in the transparency of safety risk information for Chinese listed pharmaceutical enterprises.

Indexes and scores for the level between “relatively good” and “good”	
*C*_22_	0.8179
*C*_63_	0.8173
*C*_21_	0.8056

**Table 14:** Indexes and scores for the level between “general” and “relatively good” in the transparency of safety risk information for Chinese listed pharmaceutical enterprises.

Indexes and scores for the level between “general” and “relatively good”	
*C*_24_	0.7892
*C*_72_	0.7751
*C*_84_	0.7744
*C*_62_	0.7682
*C*_64_	0.7616
*C*_41_	0.7595
*C*_83_	0.7512
*C*_42_	0.7495
*C*_16_	0.7483
*C*_34_	0.7339
*C*_52_	0.7274
*C*_91_	0.7165
*C*_32_	0.7059
*C*_31_	0.7042
*C*_92_	0.6897
*C*_94_	0.6758
*C*_15_	0.6678
*C*_81_	0.7787
*C*_61_	0.7750
*C*_11_	0.7715
*C*_82_	0.7675
*C*_33_	0.7600
*C*_53_	0.7531
*C*_86_	0.7505
*C*_51_	0.7484
*C*_54_	0.7365
*C*_43_	0.7282
*C*_85_	0.7219
*C*_23_	0.7092
*C*_13_	0.7051
*C*_93_	0.6970
*C*_14_	0.6782
*C*_74_	0.6697
*C*_73_	0.6634

**Table 15:** Indexes and scores for the level between “relatively poor” and “general” in the transparency of safety risk information for Chinese listed pharmaceutical enterprises.

Indexes and scores for the level between “relatively poor” and “general”	
*C*_44_	0.5971
*C*_71_	0.5634
*C*_12_	0.5587

## Data Availability

The data used to support the findings of this study are available from the corresponding author upon request.
